# Prevalence of Triglyceride–Glucose Index-Defined Insulin Resistance and Its Association with Recorded Cardiovascular Disease Among Adults in the Municipality of Naples: A Cross-Sectional Primary-Care Study

**DOI:** 10.3390/jcm15218244

**Published:** 2026-10-23

**Authors:** Serafino Fazio, Vincenzo Santagada, Gaetano Piccinocchi, Giuseppe Signoriello, Rosanna Ortolani, Ilaria Loperto, Flora Affuso, Roberto Piccinocchi, Ilenia Lorenza Calcaterra, Matteo Di Minno

**Affiliations:** 1 School of Medicine, Federico II University, via S. Pansini 5, 80131 Naples, Italy; ilenialorenza.calcaterra@unina.it; 2 Dipartimento di Farmacia, Università Federico II, Assessorato alla Salute e Verde, Comune di Napoli, 80133 Naples, Italy; santagad@unina.it; 3 Società Italiana di Medicina Generale, Via del Sansovino, 50142 Florence, Italy; piccinocchi.gaetano@simg.it; 4 University of Campania “Luigi Vanvitelli”, 80100 Naples, Italy; giuseppe.signoriello@gmail.com; 5 Epidemiology, Prevention and Cancer Registry Unit, ASL Napoli 1 Centro, Via Comunale del Principe, 80125 Naples, Italy; rosanna.ortolani@aslnapoli1centro.it (R.O.); ilaria.loperto@aslnapoli1centro.it (I.L.); 6 Independent Researcher, 80129 Nalles, Italy; flora.affuso70@gmail.com; 7 AOU-Azienda Ospedaliera Universitaria “Luigi Vanvitelli”, 80100 Naples, Italy; roberto.piccinocchi@gmail.com

**Keywords:** insulin resistance, TyG, risk factor, prevalence, cardiovascular disease

## Abstract

**Objectives**: Assess the prevalence of insulin resistance (IR) in the adult population of the municipality of Naples in Italy and its association with cardiovascular diseases. Methods: We performed a cross-sectional analysis of adults aged 18–60 years without recorded diabetes who were followed by general practitioners affiliated with the COMEGEN cooperative, ASL Napoli 1 Centro. Participants were included if concurrent fasting glucose and triglyceride values from the preceding six months were available to calculate the triglyceride–glucose index (TyG), as a surrogate index of IR. IR was defined using an operational cut-off of TyG ≥ 8.5. **Results**: Of 17,510 selected records, 17,481 had calculable TyG; 5707 met the IR criterion (32.65%; 95% CI 31.96–33.35%). Recorded cardiovascular conditions were present in 44.98% with IR and 28.90% without IR. The adjusted odds ratio (OR) for IR was 1.252 (95% CI 1.137–1.379; *n* = 9866), and the adjusted prevalence ratio (PR) was 1.133 (95% CI 1.079–1.190). Adding IR to a model containing age, sex, and BMI increased the ROC AUC from 0.7438 to 0.7452 (*p* = 0.0477), a negligible absolute gain. **Conclusions**: TyG-defined IR was common and associated with recorded cardiovascular conditions, but offered little incremental discrimination; causality cannot be inferred from these cross-sectional data. However, the high prevalence of IR, associated with cardiometabolic risk, should prompt health institutions to inform the public about the existence of IR, a condition associated with an increased cardiometabolic risk that could be counteracted through healthier lifestyles, and encourage the scientific community to conduct prospective randomized controlled trials to determine whether early screening for IR, and subsequent intervention to counteract it, can yield benefits for cardiovascular outcomes.

## 1. Introduction

Insulin resistance (IR), accompanied by compensatory hyperinsulinemia, precedes type 2 diabetes by several years and is mechanistically linked to a broad cardiometabolic phenotype that includes dysglycemia, atherogenic dyslipidemia, visceral adiposity, hypertension, endothelial dysfunction, a prothrombotic state, and subclinical target organ damage [1,2,3,4,5,6,7,8]. IR and compensatory hyperinsulinemia have also been implicated in cellular senescence, neurodegenerative disease, and cancer biology [9,10,11,12,13,14,15,16]. Although the euglycemic–hyperinsulinemic clamp remains the reference standard for quantifying insulin sensitivity, its complexity, cost, and time requirements preclude its use for large-scale population screening [1].

The prevalence of IR appears to be increasing in the city of Naples, just as is happening worldwide, in parallel with the global burden of type 2 diabetes and related metabolic conditions, although estimates vary substantially by population, definition, and ascertainment strategy [17,18,19,20,21,22,23,24]. Genetic determinants may contribute to selected IR phenotypes [25,26], but at the population level, the predominant modifiable drivers include sedentary behavior, adverse dietary patterns, excess caloric intake, and visceral adiposity [27,28,29,30]. This context is particularly relevant to Italy and to Naples, where childhood overweight, obesity, and insulin resistance are also on the rise, and where cardiovascular disease remains a major public health burden [31,32,33,34,35,36].

Several surrogate indices have therefore been proposed. Among them, the triglyceride–glucose (TyG) index is attractive for epidemiological and primary-care settings because it requires only fasting triglyceride and glucose values, variables that are routinely available and inexpensive to obtain [37,38,39,40,41,42,43,44]. However, TyG should be regarded as a proxy for IR rather than a diagnostic equivalent of clamp-measured insulin resistance; cut-off values vary by population, age, sex, adiposity, ethnicity, and clinical context [45,46,47]. Furthermore, important confounders such as lipid-lowering therapy, antihypertensive therapy, smoking, socioeconomic status, physical activity, diet, or access to care are likely relevant to both TyG and CVD.

The municipality of Naples is characterized by marked heterogeneity in socioeconomic conditions, lifestyle exposures, obesity burden, and access to preventive care. These factors are plausibly relevant to the distribution of IR and downstream cardiovascular risk [48,49]. The hypothesis is that IR accompanied by hyperinsulinemia is an independent risk factor for numerous diseases—particularly CVD, with a prevalence that varies by regions—and that it must be identified promptly, well before the onset of type 2 diabetes and irreversible damage, to enable information and preventive treatment. The present study was designed to estimate the prevalence of IR, as assessed by the TyG ≥ 8.5, among adults aged 18–60 years living in the municipality of Naples and followed in the COMEGEN general-practice network. The secondary objectives were to describe age- and district-specific variation and to evaluate the cross-sectional association between TyG-defined IR and recorded cardiovascular disease (CVD).

The study originated from a collaboration among the TDC19 ETS foundation, the Municipality of Naples, the ASL Napoli 1 Centro (Local Health Authority), the University of Naples Federico II, and the COMEGEN general practitioner cooperative.

The study was registered on ClinicalTrials.gov under number NCT07785518.

## 2. Materials and Methods

### 2.1. Study Design, Setting, and Data Source

This was a cross-sectional observational study based on routinely collected primary-care data. The data source was the COMEGEN Cooperative database. This Cooperative includes general practitioners affiliated with ASL Napoli 1 Centro, and the database contains clinical and laboratory information for more than 200,000 individuals distributed across municipal districts of Naples. In the database, there were 123,752 white-race citizens aged 18–60 years. The race of each subject was recorded in the database, and, to avoid introducing further bias, only white-race citizens were included in the analysis.

For the present analysis, in this large sample, the most recent available records within the six months preceding data extraction were screened. Participants were eligible if they were aged 18–60 years, had no recorded overt diabetes, and had fasting glucose and fasting triglyceride values obtained at the same time within the preceding six months, allowing TyG calculation. The glucose and triglyceride levels were measured at the same time in each individual, though not on the same day for all subjects. However, for everyone, it fell within the preceding 6-month period. The data extraction date was 19 February 2026.

### 2.2. Participants and Sampling

The analytic cohort was a convenience sample of adults aged 18–60 years of white race, with the laboratory measurements required for TyG calculation. It should not be interpreted as a probability sample of Naples residents. The availability of laboratory testing, health-care access, pharmacologic therapy, comorbidity, and socioeconomic characteristics may have influenced the selection.

### 2.3. Patients and Public Involvement

Patients and the public were not involved in this research.

### 2.4. Variables and Definitions

Demographic, anthropometric, biochemical, clinical, and geographic variables were extracted from the COMEGEN primary-care database. The demographic variables included age, defined as age at the moment of index laboratory assessment, and sex, as recorded in the general-practitioner database. The anthropometric data included body mass index (BMI), calculated as weight in kilograms divided by height in meters squared. The biochemical variables included fasting plasma glucose, triglycerides, total cholesterol, high-density lipoprotein cholesterol, and low-density lipoprotein cholesterol, all expressed in mg/dL. All the laboratories used, to quantify triglycerides and glucose, employ automated, highly standardized enzymatic assays with universal reporting units, with comparable normal ranges. The triglyceride–glucose index was calculated using fasting triglyceride and fasting glucose values according to the prespecified formula: TyG = ln[(fasting triglycerides (mg/dL) × fasting glucose (mg/dL))/2]. We considered the recorded values to be fasting levels because the general practitioners in this cooperative advise their patients to have the blood sample taken after fasting for at least 12 h. The value TyG ≥ 8.5 was considered an operational cut-off for the definition of IR. This value was suggested by some reports in the scientific literature on the topic [50,51,52,53,54,55,56,57,58,59,60] and by the evaluation of TyG values in 500 subjects (aged from 18 to 60 years, well-balanced across age decades and sex) under our care with a calculated HOMA-IR indicative of IR (>2.5), for whom it was also possible to simultaneously calculate the TyG—which, in these subjects, was consistently found to be ≥8.5 (data not published). However, this analysis cannot establish the discriminatory performance of the TyG cutoff without a non-insulin-resistant comparison group. It is well known that TyG values depend on the age, sex, and race of the subject being evaluated [55,60]; consequently, extensive confirmatory studies are needed to verify whether a TyG value ≥ 8.5 can be sufficiently accurate to indicate insulin resistance in the Italian white population. Nevertheless, there is a substantial body of literature addressing this topic. Online sources report that TyG values ≥ 8.5 generally indicate insulin resistance and correlate reasonably well with HOMA-IR and the diagnosis of metabolic syndrome [53]. It has been reported that, according to ROC analysis, the optimal TyG cut-off point for NAFLD was 8.5, with an area under the ROC curve (AUC) of 0.782 (95% CI 0.773–0.790) and sensitivity and specificity of 72.2% and 70.5%, respectively [54]. The relationship between IR and nonalcoholic fatty liver disease (NAFLD)—now more formally termed metabolic dysfunction-associated fatty liver disease (MAFLD) or MASLD—is bidirectional, with one acting as both a culprit and a consequence in a self-reinforcing vicious cycle [55,56]. Furthermore, given that the TyG index cut-off value can be influenced by age, sex, and race, a study estimated the TyG cut-off value for discriminating insulin resistance based on the previously established HOMA-IR cut-off. There is significant statistical and clinical concordance between the HOMA-IR index and the TyG (triglyceride–glucose) index in identifying insulin resistance (IR). Both biomarkers show a strong linear correlation with the development of metabolic syndrome, type 2 diabetes, and cardiovascular risk [57,58].

The clinical information for the subjects enrolled in the study included the presence of recorded cardiovascular disease, as documented in the primary-care records at the time when fasting blood glucose and triglyceride levels were evaluated. Recorded cardiovascular disease is defined as diseases of the circulatory system according to the International Classification of Diseases, 9th Revision (ICD-9), excluding acute rheumatic diseases (ICD 390-392), but including arterial hypertension. Thus, this encompasses all circulatory system diseases from ICD 393 to ICD 459.

Arterial hypertension was included—despite being considered more of a risk factor than a disease—because numerous studies have found it to be closely associated with insulin resistance, and because it is classified as a disease in the ICD-9. In Italy, the National Health System still uses ICD-9 to classify diseases.

The analytic dataset contained a composite indicator of recorded cardiovascular conditions but did not provide separate individual-level hypertension indicators. Consequently, an analysis excluding hypertension from the composite outcome could not be performed; the outcome should not be interpreted as incident or exclusively atherosclerotic cardiovascular disease.

The clinical outcome was a binary indicator of cardiovascular conditions recorded in primary-care records, including arterial hypertension. Thus, it does not specifically represent incident cardiovascular events, major adverse cardiovascular events, or exclusively atherosclerotic cardiovascular disease. Geographic information was used for exploratory descriptive analyses.

### 2.5. Statistical Analysis

Continuous variables are summarized as mean ± standard deviation (SD) or median and interquartile range (IQR), depending on the distributional characteristics. The distributions were assessed by graphical inspection and, where appropriate, formal normality testing; given the large sample size, clinical relevance and distributional shape were prioritized over normality-test *p*-values alone.

For methodological consistency and reproducibility, the TyG index was recalculated from the recorded fasting triglyceride and glucose values using the predefined formula. Patients with missing values for either component were excluded from TyG-based analyses, even if a manually calculated TyG value had previously been entered into the database.

To evaluate potential selection associated with missing BMI, age- and sex-adjusted models were compared in all 17,481 participants with calculable TyG (OR 1.496, 95% CI 1.392–1.607) and in the 9866 BMI-complete participants (OR 1.472, 95% CI 1.341–1.616). Multiple imputation was not undertaken; these comparisons do not establish a missing-at-random mechanism or exclude selection bias.

The analytic dataset did not include a general-practitioner or practice identifier; therefore, clustering within general practitioners or practices was not modeled and standard errors were not cluster-adjusted.

Age and sex were selected a priori as epidemiologically relevant covariates related to metabolic status and recorded cardiovascular morbidity. BMI was included to assess the association conditional on adiposity; as adiposity may also lie on relevant causal pathways, the BMI-adjusted association should not be interpreted as a causal direct effect. Covariate selection was not based solely on univariable statistical significance.

Continuous variables are summarized as mean (SD) or median (IQR), as appropriate, and categorical variables as count and percentage. Prevalence estimates and their 95% confidence intervals were determined using the Wilson score method. Age-stratified analyses were descriptive; the denominators for TyG-specific summaries exclude records without calculable TyG.

Recorded cardiovascular conditions were the binary outcome, and TyG-defined IR was the main exposure. Logistic regression was used to estimate unadjusted and age-/sex-adjusted ORs in the TyG-valid sample (*n* = 17,481), and age-/sex-/BMI-adjusted ORs in complete cases (*n* = 9866). Modified Poisson regression with robust standard errors was used to estimate the corresponding prevalence ratios (PRs). To isolate the contribution of BMI adjustment from complete-case selection, age-/sex-adjusted and age-/sex-/BMI-adjusted models were additionally fitted on the same 9866 complete cases. TyG was also modeled continuously. ROC curves for models with age, sex, and BMI, with and without IR, were compared on the same complete cases using roccomp (tests of equality of ROC areas). BMI was unavailable for 7628/17,510 (43.56%) records; 29/17,510 had noncalculable TyG. All associations are cross-sectional.

Age and sex were prespecified as epidemiologically relevant covariates, and BMI was included to assess the association conditional on overall adiposity. Models were not adjusted for clustering by general practitioner because practice identifiers were unavailable. For geographic analysis, linkage was based on agreement across eight recorded variables; exact matches agreed on all eight and partial matches on seven. Sensitivity analyses excluded partially matched records.

### 2.6. Sample Size and Ethics

The minimum required sample size for estimating an expected IR prevalence of 30% with 1% absolute precision at a 95% confidence level was 8068 participants. The final analytic sample of 17,510 participants was therefore adequate for prevalence estimation, although the representativeness depends on the completeness and selection mechanisms of available laboratory data.

Physicians obtained informed consent from patients for the anonymized use of their data. The study was approved by the Campania I Territorial Ethics Committee on 20 January 2026, and was conducted in accordance with the principles of the Declaration of Helsinki.

## 3. Results

In the database source, there were 123,752 individuals aged 18–60 years. Of these, 6853 were excluded due to the presence of diabetes and 99,389 because they did not have simultaneous blood glucose and triglyceride measurements within the preceding six months. Therefore, 17,510 (Table 1) non-diabetic participants had recent concurrent glucose and triglyceride data and were included in the complete-case analysis. The analytic cohort had a mean age of 45.5 ± 11.7 years, and 9954 participants (56.8%) were women. The baseline characteristics by age group are shown in Table 1.

**Figure 1:**
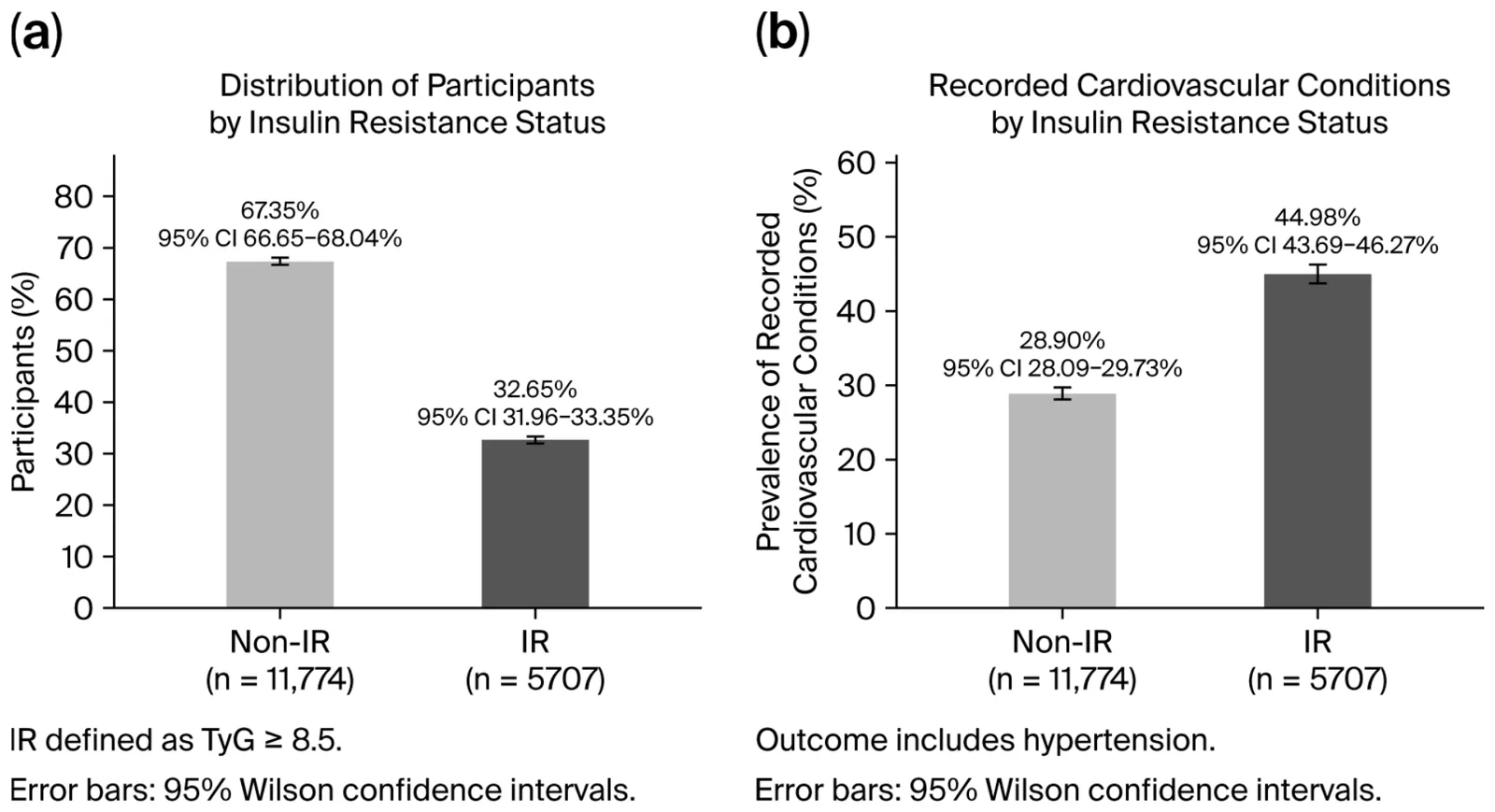
(**a**) Distribution of participants by TyG-defined insulin resistance status (*n* = 17,481). The non-IR group is shown in light gray, and the IR group in dark gray, using the same grayscale convention in (**b**) Prevalence of cardiovascular conditions according to the presence or not of IR. Error bars represent 95% Wilson confidence intervals.

TyG was calculable for 17,481 of the 17,510 selected participants; 5707 had TyG-defined IR (32.65%, 95% CI 31.96–33.35%) (Figure 1). The mean recalculated TyG was 8.30 (SD 0.53). IR prevalence increased by age group: 444/2632 (16.87%) at 18–30 years, 747/2772 (26.95%) at 31–40 years, 1448/4304 (33.64%) at 41–50 years, and 3068/7773 (39.47%) at 51–60 years.

Compared with participants without IR, those with IR had higher mean BMI (27.94 vs. 25.02 kg/m^2^), fasting glucose (94.80 vs. 86.58 mg/dL), triglycerides (167.98 vs. 72.94 mg/dL), total cholesterol (200.58 vs. 178.20 mg/dL), and LDL cholesterol (120.63 vs. 105.60 mg/dL), and lower HDL cholesterol (48.70 vs. 58.44 mg/dL). Recorded cardiovascular conditions occurred in 2567/5707 participants with IR (44.98%) and 3403/11,774 without IR (28.90%) (Figure 1). These are cross-sectional comparisons.

To facilitate interpretation of the descriptive findings, Figure 1 compares the prevalence of recorded cardiovascular conditions, including hypertension, between participants with and without TyG-defined IR. The prevalence was 44.98% (2567/5707; 95% CI 43.69–46.27%) in the IR group and 28.90% (3403/11,774; 95% CI 28.09–29.73%) in the non-IR group. The error bars show 95% Wilson confidence intervals. This unadjusted comparison complements, but does not replace, the adjusted regression analyses.

### 3.1. Multivariable Analysis

**Figure 2:**
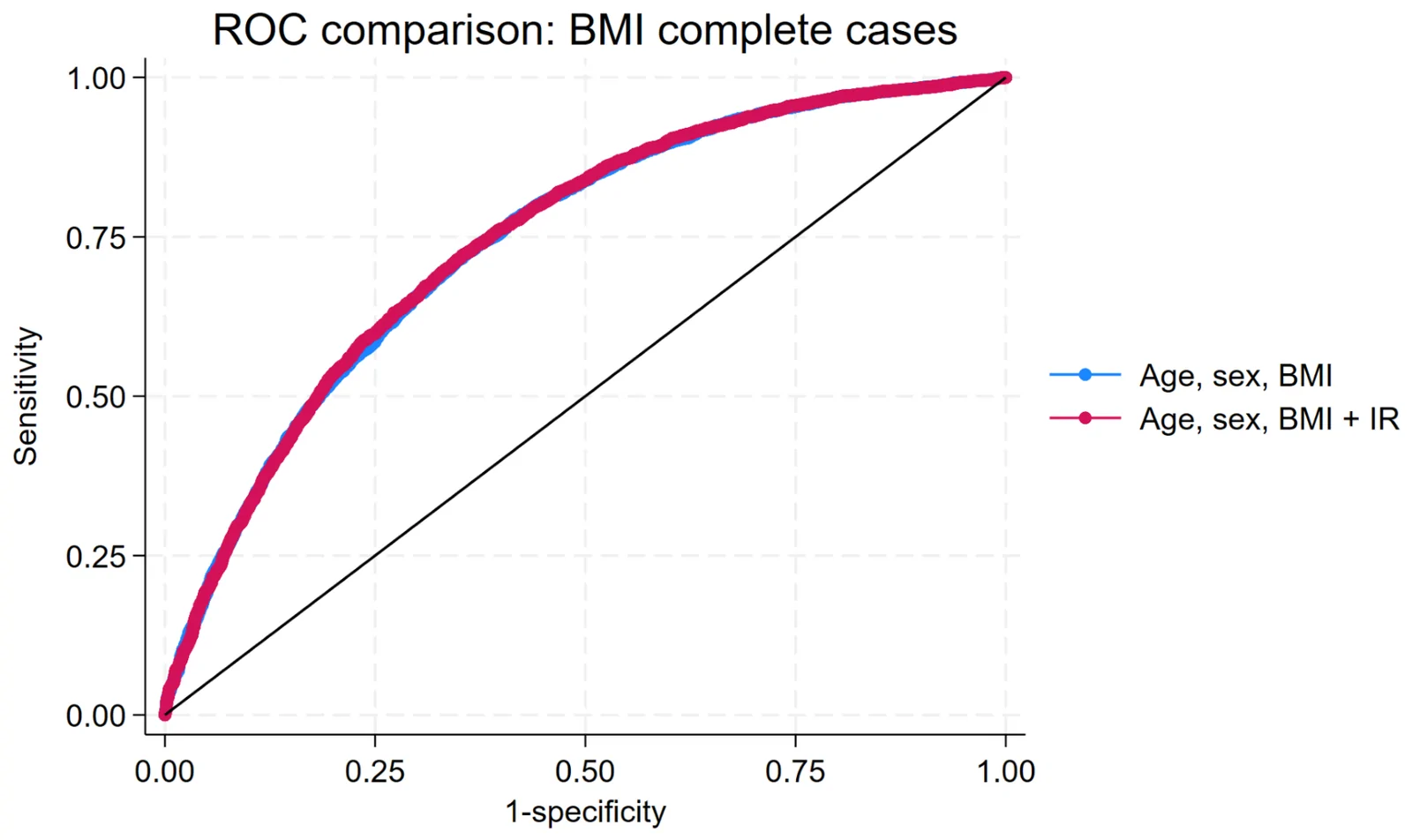
ROC curves for the base model (age, sex, BMI) and extended model (+ TyG-defined IR); *n* = 9866.

In 17,481 participants with calculable TyG, the unadjusted OR for recorded cardiovascular conditions comparing IR with non-IR was 2.011 (95% CI 1.883–2.147). The age-/sex-adjusted OR was 1.496 (95% CI 1.392–1.607), and the robust Poisson PR was 1.250 (95% CI 1.202–1.300). Among the same 9866 complete cases, the age-/sex-adjusted OR was 1.472 (95% CI 1.341–1.616), compared with 1.252 (95% CI 1.137–1.379) after additional BMI adjustment; the corresponding PRs were 1.230 (95% CI 1.171–1.292) and 1.133 (95% CI 1.079–1.190). Thus, the attenuation after BMI adjustment was not attributable solely to a change in sample size. When modeled continuously, TyG had an adjusted OR of 1.400 per unit (95% CI 1.277–1.535). In ROC analysis (Figure 2) of these 9866 complete cases, the model containing age, sex, and BMI had an AUC of 0.7438 (95% CI 0.7340–0.7537), and adding IR increased the AUC to 0.7452 (95% CI 0.7354–0.7550); χ^2^(1) = 3.92, *p* = 0.0477. Despite nominal statistical significance, the absolute AUC difference was only 0.0014 and is unlikely to represent a clinically meaningful gain in discrimination.

These estimates should be interpreted as cross-sectional associations (Table 2). They do not establish that IR caused CVD. For a causal or prognostic claim, longitudinal follow-up or a clearly specified model with incident CVD as the outcome would be required.

Geographic linkage used agreement across eight demographic, anthropometric, and laboratory fields. Among the linked records, 758 had partial agreement (seven of eight fields), of which 731 had calculable TyG and contributed to the geographic analysis. A sensitivity analysis restricted to exact eight-field matches included 15,078 participants, 4855 of whom had TyG-defined IR (32.20%, 95% CI 31.46–32.95%), compared with 5120/15,809 (32.39%) when partial matches were included. The difference was 0.19 percentage points, and district-specific absolute differences were below one percentage point. District-to-municipality labels remain subject to confirmation against the administrative code legend.

### 3.2. Geographic Distribution by Municipality

The exploratory prevalence of IR in the different districts of Naples ranged from 27.3% in the Chiaia-Posillipo district to 37.2% in the Scampia-Chiaiano district. The two districts are known to differ significantly, on average, in terms of socioeconomic, health and cultural development (Table 3). The exploratory geographic analysis included 15,809 participants with valid recalculated TyG values and assigned DSB, including 731 partial matches with valid TyG. After excluding these partial matches, 15,078 exact-matched participants remained; IR prevalence was 32.20% (4855/15,078), compared with 32.39% (5120/15,809) in the full linked sample. District-specific absolute prevalence differences were below one percentage point. These results are descriptive and depend on the validity of the geographic linkage and distribution mapping.

Correction to the opening prevalence statement: The verified prevalence of TyG-defined IR in the analytic cohort with calculable TyG was 5707/17,481 (32.65%; 95% CI 31.96–33.35%), rather than the earlier archived estimate of 31.0%. The recalculated TyG mean was 8.30 (SD 0.53). This estimate describes a selected primary-care sample and is not population-representative.

## 4. Discussion

In this large cross-sectional primary-care sample from the municipality of Naples, 32.65% (5707/17,481) of non-diabetic adults aged 18–60 years were classified as insulin-resistant using the operational threshold TyG ≥ 8.5. This estimate is within the broad range reported in contemporary epidemiological studies [35,61].

This estimate refers to a selected primary-care population with available fasting glucose and triglyceride measurements, rather than to a probability sample representative of all Naples residents [17,18,19,20,21,22,23,24]. Residual confounding by medications, smoking, diet, physical activity, and socioeconomic circumstances cannot be excluded. Prospective population-based studies are needed to assess the clinical utility of TyG screening and whether interventions targeting insulin resistance improve cardiovascular outcomes. However, three meta-analyses have shown that the TyG index can be considered a promising biomarker for screening and predicting various medical conditions, particularly those related to insulin resistance and metabolic disorders [62,63,64]. One meta-analysis included 32 studies and revealed significant associations among the TyG index and various health outcomes, in particular, increased risk of type 2 diabetes mellitus, gestational diabetes, chronic kidney disease, and diabetic retinopathy. Furthermore, the index was significantly higher in patients with obstructive sleep apnea, metabolic syndrome and nonalcoholic fatty liver disease, and was significantly associated with increased risk of heart failure, atrial fibrillation and hypertension [62]. Another meta-analysis, based on a total of 85 studies, concluded that TyG-based obesity composite indices were associated with many cardiovascular outcomes and mortality risk, and that they could be used in addition to traditional risk assessment for risk stratification and primary prevention. However, the authors said that multicenter prospective studies are needed to further validate the causal associations and the usefulness of these indices across different populations [63]. The third meta-analysis was performed to assess the association between TyG, as surrogate index of IR, and the presence of atrial fibrillation (AF). The conclusion of this study was that, although further clinical studies are needed to demonstrate the possibility of its use for routine clinical assessment and screening tool, the results showed that TyG was significantly higher in patients with AF than non-AF subjects [64]. Although there is a large body of literature supporting the use of TyG as a surrogate index of IR and the association of TyG with numerous cardiovascular diseases, this is not yet sufficient to demonstrate a causal relationship.

The observed phenotype was coherent with the biology of IR: participants classified as insulin-resistant by TyG had higher BMI, fasting glucose, triglycerides, total cholesterol, and LDL cholesterol, and lower HDL cholesterol. Recorded CVD was also substantially more common in the TyG-defined IR group. The interpretation of the cardiovascular findings requires caution because the temporal relationship between TyG-defined IR and cardiovascular disease cannot be determined. However, the association with the presence of cardiovascular disease is significantly higher in the subjects with TyG ≥ 8.5 than in the subjects with TyG < 8.5. These findings support the concept that TyG-defined IR clusters with established cardiometabolic risk factors, although the present analysis cannot determine whether IR preceded CVD, whether CVD and its treatments influenced metabolic markers, or whether both reflect shared upstream determinants such as adiposity, diet, physical inactivity, socioeconomic deprivation, and access to preventive care [1,2,3,4,5,6,7,8,27,28,29,30,43,44,48,49].

It is largely reported in the literature that compensatory hyperinsulinemia may contribute to vascular dysfunction, blood pressure elevation, sympathetic activation, renal sodium retention, and renin–angiotensin–aldosterone system activation [4,65,66,67,68,69,70,71,72,73,74]. It has also been linked to chronic heart failure phenotypes and left-ventricular remodeling, including subclinical remodeling in population cohorts [72,73,74]. This is clinically relevant because cardiovascular risk often becomes apparent at or before the transition to overt diabetes, and contemporary cardiometabolic guidelines emphasize aggressive risk factor management in diabetes and pre-diabetes [75]. Whether the earlier identification and treatment of IR through lifestyle intervention, insulin-sensitizing pharmacotherapy, or broader cardiometabolic approaches can reduce incident diabetes and CVD requires prospective evaluation [1,75,76,77,78].

With recorded cardiovascular conditions as the outcome, TyG-defined IR remained associated with the outcome after adjustment for age, sex, and BMI (OR 1.252, 95% CI 1.137–1.379; robust Poisson PR 1.133, 95% CI 1.079–1.190; *n* = 9866). On the identical complete-case sample, adding BMI attenuated the age-/sex-adjusted association (OR 1.472 to 1.252; PR 1.230 to 1.133). This comparison does not resolve whether BMI is a confounder, mediator, or both. Adding corrected IR to the age/sex/BMI model increased the in-sample AUC only from 0.7438 to 0.7452 (ΔAUC ≈ 0.0014), indicating negligible incremental discrimination; no external validation was performed.

The exploratory distribution of TyG-defined IR across Naples districts showed geographic heterogeneity (27.3–37.2% among assigned districts). Individual socioeconomic variables were not available, so these differences cannot be directly attributed to socioeconomic factors at the individual level. Moreover, the reconstructed geographic linkage and proposed DSB-to-municipality mapping need validation; territorial findings should be interpreted cautiously.

This study has several strengths: a large real-world primary-care cohort, the use of routinely available biomarkers, the exclusion of overt diabetes, the availability of age-stratified data, and district-level mapping within a large urban municipality. These features make the findings relevant for local prevention planning by information and intervention and for designing pragmatic screening pathways in primary care.

Several limitations warrant emphasis. The cross-sectional design precludes causal or temporal inference. The convenience sample was restricted to individuals with available laboratory data, and BMI was missing for 7628/17,510 selected records (43.56%). Participants with observed BMI differed from those without BMI information, so selection bias cannot be excluded despite the sensitivity analyses. The composite outcome included arterial hypertension; diagnoses were not available separately in the analytic dataset, preventing an analysis excluding hypertension. General-practitioner or practice identifiers were unavailable, and variance estimates did not account for clustering within practices. Lifestyle, medication, and socioeconomic variables were incompletely captured. The exploratory district analysis relied on reconstructed geographic linkage; excluding partial matches produced similar descriptive estimates, but the mapping and linkage require external validation. ROC discrimination was assessed in-sample without external validation.

These limitations do not invalidate the main prevalence signal, but they require a more cautious interpretation than a causal statement. The most defensible conclusion is that TyG-defined IR, assessed by an operational cutoff value of TyG ≥ 8.5, is common in this primary-care population and is associated with a higher prevalence of recorded CVD and an unfavorable cardiometabolic profile. This supports targeted education, lifestyle intervention, and prospective evaluation rather than immediate claims that TyG screening, as surrogate index of IR, will reduce CVD outcomes.

## 5. Conclusions

In a large primary-care cohort of non-diabetic adults aged 18–60 years from the municipality of Naples, approximately one third had TyG ≥ 8.5, suggesting possible insulin resistance. Values of TyG ≥ 8.5 were associated with older age, male sex, higher BMI, and a higher prevalence of recorded cardiovascular disease. These findings should urge health institutions to raise awareness about IR and its pathological implications among the poorly informed public—explaining that it can be easily countered through healthy lifestyle choices—and encourage the scientific community to conduct randomized controlled prospective studies to determine whether promptly addressing IR (identified via surrogate indices such as TyG) yields benefits for cardiovascular outcomes.

## Figures and Tables

**Table 1:** Baseline characteristics of the analytic cohort by age group.

Factor	Total (*n* = 17,510)	18–30 (*n* = 2635)	31–40 (*n* = 2776)	41–50 (*n* = 4313)	51–60 (*n* = 7786)	*p*-Value
Age, mean (SD)	45.5 (11.7)	24.6 (3.6)	35.8 (2.8)	46.1 (2.9)	55.8 (2.9)	<0.001
Sex, *n* (%)						<0.001
Female	9954 (56.8%)	1387 (52.6%)	1507 (54.3%)	2511 (58.0%)	4549 (58.4%)	
Male	7556 (43.2%)	1248 (47.4%)	1269 (45.7%)	1802 (41.8%)	3237 (41.6%)	
BMI, mean (SD)	26.0 (5.5)	24.0 (5.8)	25.5 (5.7)	26.3 (5.7)	26.5 (5.1)	<0.001
Glucose, mean (SD)	89.0 (11.8)	84.6 (10.0)	87.0 (10.8)	89.0 (11.3)	91.7 (12.3)	<0.001
Total cholesterol, mean (SD)	185.5 (39.0)	159.5 (31.0)	175.4 (35.0)	188.9 (36.7)	196.0 (39.3)	<0.001
HDL cholesterol, mean (SD)	55.3 (15.6)	52.6 (13.3)	53.4 (14.7)	55.3 (15.9)	56.8 (16.2)	<0.001
LDL cholesterol, mean (SD)	110.5 (34.0)	90.5 (27.0)	103.5 (31.0)	113.7 (32.5)	118.1 (35.0)	<0.001
Triglycerides, mean (SD)	104.0 (66.0)	85.7 (53.9)	97.6 (60.6)	107.7 (75.6)	110.3 (64.8)	<0.001
TyG index, mean (SD)	8.30 (0.53)	8.07 (0.47)	8.21 (0.52)	8.32 (0.55)	8.40 (0.51)	<0.001
Recorded CVD, *n* (%)						<0.001
Yes	5983 (34.2%)	218 (8.3%)	457 (16.5%)	1358 (31.5%)	3950 (50.7%)	
No	11,527 (65.8%)	2417 (91.7%)	2319 (83.5%)	2955 (68.5%)	3836 (49.3%)	

Abbreviations: BMI, body mass index; CVD, recorded cardiovascular conditions (including hypertension); HDL, high-density lipoprotein; LDL, low-density lipoprotein; SD, standard deviation; TyG, triglyceride–glucose index. Table 1 describes the selected cohort; TyG-based results are restricted to the 17,481 participants with calculable TyG.

**Table 2:** Association of recalculated TyG-defined insulin resistance with recorded cardiovascular conditions: logistic and robust Poisson regression models.

Model/Effect of IR	Estimate	95% CI	*p*-Value
Logistic, unadjusted (*n* = 17,481); OR	2.011	1.883–2.147	<0.001
Logistic, age + sex (*n* = 17,481); OR	1.496	1.392–1.607	<0.001
Logistic, age + sex (*n* = 9866); OR	1.472	1.341–1.616	<0.001
Logistic, age + sex + BMI (*n* = 9866); OR	1.252	1.137–1.379	<0.001
Robust Poisson, age + sex (*n* = 17,481); PR	1.250	1.202–1.300	<0.001
Robust Poisson, age + sex (*n* = 9866); PR	1.230	1.171–1.292	<0.001
Robust Poisson, age + sex + BMI (*n* = 9866); PR	1.133	1.079–1.190	<0.001
Logistic, continuous TyG per +1 (*n* = 9866); OR	1.400	1.277–1.535	<0.001

Note: The outcome recorded cardiovascular conditions, including hypertension. IR is defined by recalculated TyG ≥ 8.5. OR, odds ratio; PR, prevalence ratio; CI, confidence interval; BMI, body mass index. The age- and sex-adjusted models use *n* = 17,481; models involving BMI use *n* = 9866 complete cases. To assess the effect of adding BMI, age/sex and age/sex/BMI estimates should be compared within the same *n* = 9866 cases.

**Table 3:** Exploratory prevalence of recalculated TyG-defined insulin resistance by Naples municipality (linked territorial sample, *n* = 15,809).

Municipality/Area	DSB	Valid *n*	IR Yes	IR No	IR (%) [95% CI]
1. Chiaia Posillipo	24	1121	306	815	27.3 [24.8–30.0]
2. Centro storico	31	1113	395	718	35.5 [32.7–38.3]
3. Stella San Carlo	29	936	307	629	32.8 [29.9–35.9]
4. San Lorenzo Vicaria	33	965	319	646	33.1 [30.2–36.1]
5. Vomero Arenella	27	3386	1004	2382	29.7 [28.1–31.2]
6. Ponticelli Barra	32	148	48	100	32.4 [25.4–40.3]
7. Miano Secondigliano	30	222	80	142	36.0 [30.0–42.5]
8. Scampia Chiaiano	28	1682	626	1056	37.2 [34.9–39.6]
9. Soccavo Pianura	26	1967	674	1293	34.3 [32.2–36.4]
10. Fuorigrotta Bagnoli	25	4269	1361	2908	31.9 [30.5–33.3]
Total		15,809	5120	10,689	32.4 [31.7–33.1]

Note: Geographic analysis was restricted to 15,809 participants with calculable TyG and available municipality-level information; 5120 had IR. DSB codes were assigned to the corresponding municipality as specified in the geographic tabulation. Confidence intervals were calculated using the Wilson method.

## Data Availability

The data used in the study were obtained from the COMEGEN database in Naples. The use of data not covered by the current ethics approval will require additional approval before being made available. Anyone seeking to access the data will need to contact the lead investigator or Dr. Piccinocchi G.

## Abbreviations

BMI, body mass index; CI, confidence interval; CVD, cardiovascular disease; GP, general practitioner; HDL, high-density lipoprotein; HI, hyperinsulinemia; IR, insulin resistance; LDL, low-density lipoprotein; OR, odds ratio; SD, standard deviation; TyG, triglyceride–glucose index.
